# Aerosol jet printing of piezoelectric surface acoustic wave thermometer

**DOI:** 10.1038/s41378-023-00492-5

**Published:** 2023-05-04

**Authors:** Nicholas McKibben, Blake Ryel, Jacob Manzi, Florent Muramutsa, Joshua Daw, Harish Subbaraman, David Estrada, Zhangxian Deng

**Affiliations:** 1 Micron School of Materials Science and Engineering, Boise State University, 1910 W University Drive, Boise, ID 83725 USA; 2 Department of Mechanical and Biomedical Engineering, Boise State University, Boise, ID 83725 USA; 3 School of Electrical Engineering and Computer Science, Oregon State University, 2500 NW Monroe Avenue, Corvallis, OR 97331 USA; 4 Idaho National Laboratory, Idaho Falls, ID 83415 USA; 5 Center for Advanced Energy Studies, Idaho Falls, ID 83401 USA

**Keywords:** Electrical and electronic engineering, Electronic properties and materials

## Abstract

Surface acoustic wave (SAW) devices are a subclass of micro-electromechanical systems (MEMS) that generate an acoustic emission when electrically stimulated. These transducers also work as detectors, converting surface strain into readable electrical signals. Physical properties of the generated SAW are material dependent and influenced by external factors like temperature. By monitoring temperature-dependent scattering parameters a SAW device can function as a thermometer to elucidate substrate temperature. Traditional fabrication of SAW sensors requires labor- and cost- intensive subtractive processes that produce large volumes of hazardous waste. This study utilizes an innovative aerosol jet printer to directly write consistent, high-resolution, silver comb electrodes onto a Y-cut LiNbO_3_ substrate. The printed, two-port, 20 MHz SAW sensor exhibited excellent linearity and repeatability while being verified as a thermometer from 25 to 200 ^∘^C. Sensitivities of the printed SAW thermometer are $-96.9\times 1{0{}^{-6}}^{\circ }$C^−1^ and $-92.0\times 1{0{}^{-6}}^{\circ }$C^−1^ when operating in pulse-echo mode and pulse-receiver mode, respectively. These results highlight a repeatable path to the additive fabrication of compact high-frequency SAW thermometers.

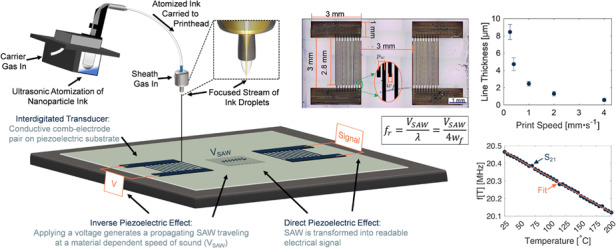

## Introduction

Piezoelectric materials output an electrical signal when mechanically stressed^1^. Conversely, they change in shape when electrically activated^2^. This effect, also known as piezoelectricity, is instantaneous and highly linear^3,4^, thus enabling innovations in compact and broadband ultrasonic transducer development. Previous studies have successfully devised piezoelectric acoustic transducers that can generate and detect bulk acoustic waves^5^, Lamb waves^6^, and shear waves^7^. Surface acoustic waves (SAWs)^8^, also referred to as Rayleigh waves^9^, are of particular interest because they are non-dispersive and propagate in a single wave mode. A typical piezoelectric SAW transducer consists of interdigitated transducers (IDTs) deposited onto a piezoelectric substrate^10^. Driven by a voltage impulse, one IDT generates a SAW propagating on the surface of the substrate, which is later converted back to an electrical signal by the same or a separate IDT. By tracking the frequency drift, time-of-flight, or insertion loss of the SAW, researchers have successfully measured ambient environmental factors like temperature and pressure^11^. SAW devices can also operate as actuators to control material properties at a quantum level^12^.

Fabrication of piezoelectric SAW transducers traditionally relies on subtractive processes, including sputter deposition and photolithography^13^. These manufacturing procedures are well-established and have resulted in extremely high-frequency (GHz) SAW transducers with ultra-fine IDT resolutions (down to 7 nm)^14^. However, both the deposition and photolithography procedures are labor- and cost-intensive. Clean rooms are often necessary for the chemical etching process, which generates large volumes of hazardous waste. Production of photolithography masks is another expensive and time-consuming process that must be repeated for every iteration of an IDT design^15^. Additive manufacturing techniques that directly write IDTs onto piezoelectric substrates provide a potentially fast and economic alternative to the fabrication of moderate frequency (<100 MHz) SAW transducers, that is more environmentally friendly than traditional subtractive manufacturing processes^16^. Direct writing processes like ink jet printing (IJP) also provide improved flexibility in terms of both design customization and substrate size, factors that are typically limited by the expense of photomask generation and the size of vacuum chamber.

Aerosol jet printing (AJP), an emerging direct writing technique, offers improved line resolution (down to 10 μm) when compared to IJP^17^. In this additive manufacturing technique a nanoparticle ink is ultrasonically atomized into a dense ink slurry consisting of droplets with diameters of ~1–5 μm. Size dependence of the droplets is related to the frequency of the atomizer^18^. The aerosolized droplets are transported from the ink reservoir to the printhead using an inert carrier gas. A sheath gas is applied inside the printhead, where the stream of microdroplets is collimated and accelerated through a tapered nozzle that aerodynamically focuses the droplets into an ordered jet that impacts the surface of a substrate^18^. The deposition is non-directional, and the larger working distance, from printer tip to substrate, enables the printer to deposit material consistently onto rough and uneven surfaces^19^, even allowing for conformal printing onto non-planar substrates^20^.

AJP has been verified as a rapid prototyping technique in the fabrication of micro-electromechanical systems (MEMS)^21^, due to its high printing speed and flexibility. Aside from metallic nanoparticles^22^, AJP can deposit a variety of material types, including thermoelectrics and 2-dimensional materials like graphene^23,24^. Researchers at the Oak Ridge National Laboratory have used AJP to deposit silver IDTs onto piezoelectric LiNbO_3_ wafers, prototyping SAW transducers with various IDT configurations^25,26^. These SAW transducers exhibit a moderate fundamental frequency (<100 MHz) that is suitable for lab on a chip technology^27–29^, and applications in acoustofluidics^30,31^. But only part of these printed SAW transducers produced good linearity when measured to 50 ^∘^C, and the rest were either shorted or output weak signals due to printing inconsistency.

Ink rheology plays a significant role in AJP, dictating whether an ink is jettable and influencing other aspects of print quality^32^. Ink properties including particle size (*D*_*h*_), density (*ρ*_*i*_), viscosity (*μ*_*i*_), and surface tension (*γ*) directly affect the atomization yield and droplet size distribution of the resultant aerosol^33^. Overspray generation is dependent on droplet size^34^, with smaller droplets being more susceptible to the expansive inertial forces encountered as the microdroplets are ejected from the printer nozzle toward the substrate surface. The interplay between the inertial forces of a fluid and its viscosity, which is quantified by the Reynold’s number (Re), also influences AJP reliability, exemplified by the complex evaporation dynamics that occur during the aerodynamic focusing and impaction phases of the process^35^. Despite the fact that ink properties influence every physical process associated with AJP, rheological parameters are minimally reported in current literature. Monitoring of these properties along with AJP parameters can lead to improvements in both process stability and repeatability, key areas of research for the advancement of this manufacturing technique^36^.

Reported in this work is the AJP of a SAW thermometer consisting of silver IDTs on a LiNbO_3_ substrate. A comprehensive study was performed on the silver ink to calculate and monitor key rheological properties and elucidate printer settings. By using a relatively low print speed, single pass coverage was achieved while maintaining an acceptable line-edge quality. A proper working distance for the printer was established using the Reynold’s number and existing theoretical literature, effectively reducing the amount of overspray generated during manufacturing. The print was monitored over time to outline an ideal process window and to evaluate instrumental drift within the system. 4-point devices were manufactured to test the electrical and structural properties of the printed thin films. Ultimately, a two-port 20 MHz SAW thermometer was prototyped using AJP. The quality factor of the printed lines was calculated, and the performance of the device was experimentally validated (i.e., sensitivity and linearity) from 25 to 200 ^∘^C.

## Materials and methods

**Fig. 1:**
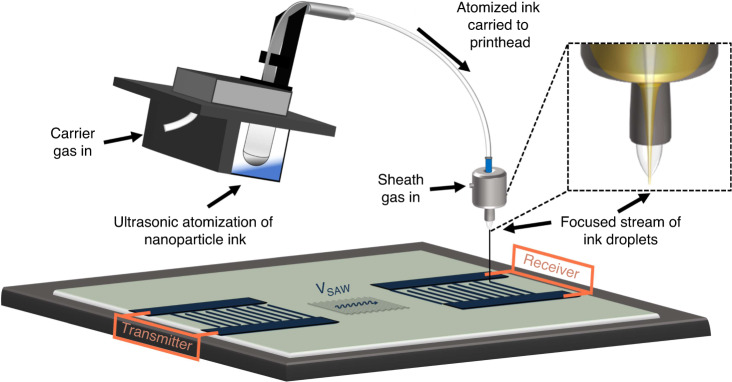
A cartoon depicting the aerosol jet printing of a SAW device. In this process, ultrasonically atomized nanoparticle ink droplets are carried from the ink reservoir to the printhead, where they are focused into an ordered stream for deposition onto the surface of a piezoelectric substrate. The SAW device functions by transforming an applied voltage into an acoustic wave that travels across the surface at a material-dependent speed of sound *V*_SAW_. Once it reaches a receiver, the acoustic wave is transformed back into a readable electrical signal

Figure 1 illustrates the Optomec Aerosol Jet 200 system used for the fabrication of compact and piezoelectric SAW thermometers. For ultrasonic atomization, the ink reservoir of the printer is filled with a nanoparticle ink. When attaching to the system, the conical vial is seated in a water bath and the ink is atomized using ultrasonic atomization. Pneumatic atomization of the ink is also possible, which has less strict limitations on nanoparticle size and viscosity, but requires considerably more initial ink volume. With intention of low-cost and small-scale prototyping, ultrasonic atomization was selected, which only requires 1–3 mL of nanoparticle ink to perform a full day of printing.

In this work, diluted Clariant TPS50 silver nanoparticle suspension was deposited onto our selected piezoelectric substrate Y-cut LiNbO_3_. A SAW device with a moderate fundamental frequency of 20 MHz was targeted due to instrument limitations. The fundamental frequency (*f*) of the SAW is directly correlated to design aspects of the device through Equation (1).

1 $$f=\frac{{V}_{{\mathrm{SAW}}}}{{l}_{w}}=\frac{{V}_{{\mathrm{SAW}}}}{4{w}_{f}}$$

In this equation, *V*_SAW_, *l*_*w*_, and *w*_*f*_ are the material-dependent speed of sound, wavelength of SAW, and electrode finger width/spacing, respectively. The IDTs were aligned along the *z*-axis of the LiNbO_3_, in which the direction of the SAW has proven to exhibit the most significant temperature dependence (${\mathtt{ \sim }}95\times 1{0{}^{-6}}^{\circ }$C^−1^)^37^. The nominal *V*_SAW_ along the *z*-axis in Y-cut LiNbO_3_ is 3488m ⋅ s^−1^ at room temperature^38^. To obtain a SAW thermometer with *f*_*r*_ ≈ 20 MHz, the desired electrode width *w*_*f*_ = 43.6 μm according to Equation (1).

### Rheology

**Fig. 2:**
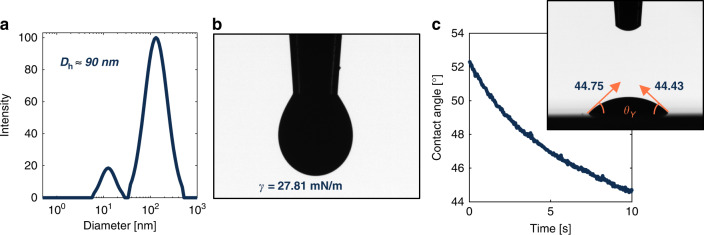
Rheological properties of a silver nanoparticle ink for AJP. **a** Dynamic light scattering spectra of silver nanoparticles suspended in water for hydrodynamic particle size measurement. **b** Image of pendant drop test measuring the surface tension of the ink. **c** Contact angle recession plot of silver nanoparticle ink on lithium niobate. Inset image shows the measurement of Young’s contact angle 10 s after deposition

Rheological characterization was first performed on the nanoparticle suspension to address the needs for ultrasonic atomization. Per AJP manufacturer recommendation, the ink particle size is limited to *D*_*h*_ < 200 nm, the viscosity of the ink is limited to *μ*_*i*_ < 10 cP, and the surface tension *γ* < 30 N ⋅ m^−1^ (ref. ^39^). To begin, concentrated Clariant TPS50 silver nanoparticle suspension was diluted with deionized (DI) water to achieve a particle loading appropriate for dynamic light scattering (DLS). DLS was performed on the dilution at room temperature using a Brookhaven Nanobrook Omni. The particle size distribution was bi-modal in nature with peaks centered around 12 and 130 nm, and the average hydrodynamic particle size of the silver nanoparticles was measured to be *D*_*h*_ ≈ 90 nm, Fig. 2a. After confirming that the particle size satisfied AJP requirements, a printable silver nanoparticle ink was synthesized by diluting the concentrated silver nanoparticle suspension 1:3 with DI H_2_O and ultrasonicating the ink mixture in a bath sonicator for 30 min. The resultant ink had a solids concentration of Φ_*i*_ = 125 mg ⋅ mL^−1^ and a measured density of *ρ*_*i*_ = 1.14g ⋅ mL^−1^. This ink mixture was selected experientially, due to the improved line quality observed during previous printing sessions, and based on existing literature^40,41^.

Dynamic viscosity (*μ*_*i*_) measurements were performed on the ink at room temperature using a Brookfield Ametek LVDVNext cone and plate rheometer. The measured viscosity of the ink, *μ*_*i*_ = 1.7 cP, is well within the limit for ultrasonic atomization. Surface tension and contact angle measurements were performed using a Biolin Scientific Theta optical tensiometer. A pendant drop test was performed on the ink, Fig. 2b, and the surface tension was measured to be *γ* = 27.81 mN ⋅ m^−1^. Aside from ensuring that ink requirements were met, the wettability of the ink-substrate system was also assessed. A sessile drop experiment was performed, in which a 5 μL droplet of the ink was deposited onto a 10 × 10 mm^2^ y-cut SAW-grade single crystal LiNbO_3_ substrate purchased from MTI Corporation, Fig. 2c. The contact angle was measured constantly to analyze the recession over time. After 10 seconds, the Young’s contact angle of the ink was determined to be *θ*_*y*_ = 44. 6^∘^ on the untreated LiNbO_3_ surface. This proved to be sufficiently low for production of high-quality printed lines, so no surface treatment was necessary for the substrates prior to printing.

### Aerosol jet printing

Optimization of the AJP process parameters was performed to enable consistent and repeatable AJP fabrication. These parameters include carrier gas flow rate (*q*_*c*_), sheath gas flow rate (*q*_*s*_), atomization current, print speed, working distance, ink bath temperature, and platen temperature. The impact of platen temperature, ink bath temperature, and atomizer current have been reported in literature^40^. However, these parameters are either system dependent or have a relatively small effect on controlling printed line width and quality. For instance, the range of atomizer current varies based on transducer health and system maintenance, and varying ink temperature effectively alters the rheological properties of the nanoparticle ink. It was determined from previous results that the print speed and focusing ratio (*F*_*R*_ = *q*_*s*_/*q*_*c*_) are primary factors that affect printed line width and quality^42^. Other parameters were chosen empirically and fixed throughout the study, Table 1.

**Table 1:** Summary of AJP parameters

Aerosol jet printer parameters		
Nozzle diameter	*d*_*n*_	150 μm
Carrier gas flow rate	*q*_*c*_	$35\,{{{{\rm{cm}}}}}^{3}{\min }^{-1}$
Sheath gas flow rate	*q*_*s*_	$55\,{{{{\rm{cm}}}}}^{3}{\min }^{-1}$
Print speed (lines)		0.25–0.40 mm ⋅ s^−1^
Print speed (pads)		0.80 mm ⋅ s^−1^
Platen temperature		70 ^∘^C
Ink temperature		20 ^∘^C
Initial ink volume		2.5 mL
Working distance		4 mm

Salary et al. has previously demonstrated AJP settings resulting in ~20 μm printed lines with high internal connectivity, low overspray amount, high line density, and good edge quality^39^. The reported settings served as an adequate starting point for determination of our AJP parameters, however, we ultimately deviated from these initial settings by decreasing the *F*_*R*_ and print speed, targeting a printed line width of ~ 40 μm. This was carried out using the settings *q*_*c*_ = $35\,{{{{\rm{cm}}}}}^{3}{\min }^{-1}$ and *q*_*s*_ = $55\,{{{{\rm{cm}}}}}^{3}{\min }^{-1}$ (*F*_*R*_ ≈ 1.6). Also, the print speed of the system was set to 0.25–0.40 mm ⋅ s^−1^ for printed lines and 0.80 mm ⋅ s^−1^ for pad filling operations. These changes appeared to have approximately doubled the width of the printed line from the reported value, estimated using the process and alignment cameras on the AJP system. The total gas flow rate of these settings is ${q}_{g}=90\,{{{{\rm{cm}}}}}^{3}{\min }^{-1}$, calculated as the sum of the *q*_*s*_ and *q*_*c*_. Based on these AJP settings the jetting velocity (*v*) and Reynold’s number were calculated using Equation (2) and Equation (3), respectively.

2 $$v=\frac{{q}_{g}}{\pi {r}_{n}^{2}}$$

3 $$Re=\frac{v{\rho }_{g}{d}_{n}}{{\mu }_{g}}=\frac{{q}_{g}{\rho }_{g}}{\pi {r}_{n}^{2}{\mu }_{g}}{d}_{n}$$

Here, *r*_*n*_ is the radius of the printer nozzle and *d*_*n*_ is the nozzle diameter, *d*_*n*_ = 150 μm. Compressed nitrogen is used as both the carrier gas and sheath gas, which has a dynamic viscosity of *μ*_*g*_ = 1.76 × 10^−5 ^kg ⋅ m^−1^s^−1^ at 300 K, and a density of *ρ*_*g*_ = 1.16 kg ⋅ m^−3^. The jetting velocity of the system was calculated to be 85 m⋅s^−1^, and the Reynold’s number to be *Re* ≈ 800, which corresponds to jet breakdown at distances greater than ~ 4 mm^43^. Therefore, the working distance of the system (from printer tip to substrate) was set to 4 mm, effectively reducing the amount of overspray generated by the onset of hydrodynamic instability.

#### Instrumental drift

**Fig. 3:**
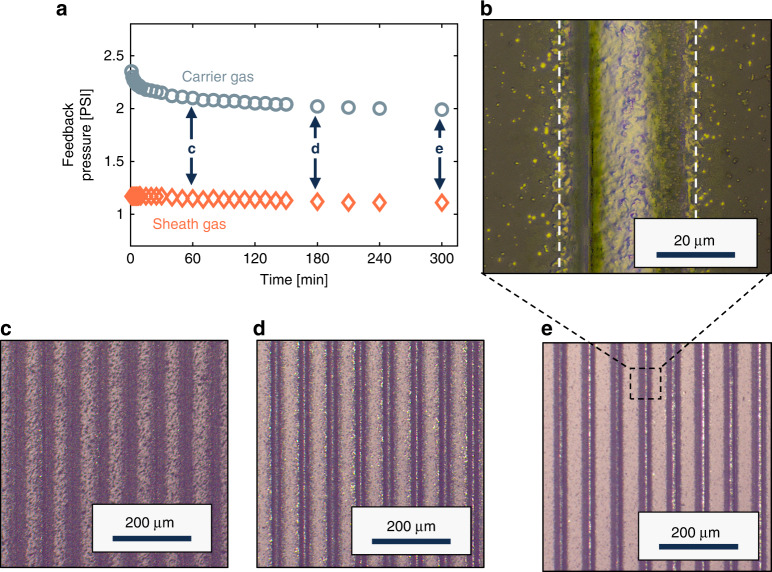
Instrumental drift and print quality evaluation for a 6-hour AJP session. **a** Carrier gas and sheath gas feedback pressures vs. time. **b** 50 × microscope image of printed lines with a measured line width of ~ 35 μm. **c**–**e** 5 × Microscope images of printed lines at (**c**) 1 h, (**d**) 3 h, (**e**) 5 h, showing an improvement in line quality and a reduction in overspray as time passed

Line quality was monitored and evaluated with respect to print time to assess the degree of instrumental drift that occurs within the system during a production run^44^. This information was used to determine the process window necessary for generation of high-quality printed lines for SAW thermometer fabrication. The feedback pressures of the carrier gas and sheath gas were recorded at constant flow rates to monitor change in gas pressures over time, Fig. 3a. Repeating serpentine lines were printed at different stages of the print session to assess how print quality changed over time during operation, Fig. 3c–e. Printed lines were imaged with a Zeiss optical microscope using the 5× and 50× objectives. Using the outlined parameters and a print speed of 0.40 mm ⋅ s^−1^ produced a line width of 35 μm, Fig. 3b.

In the early stages of production a considerable amount of overspray was observed, which decreased over the duration of the 6-h run. During the first 30 min of printing, the carrier gas feedback pressure decreased by about 10%. After ~50 min, consistent printed lines became possible, but a large degree of overspray was still present at this time. The overspray dramatically decreased after 2.5 h, and by hour-5 the printed lines were very clean and nearly void of overspray. This line quality was observed for the remaining hour of the print session. During the final hour, the print speed was further decreased to 0.25 mm ⋅ s^−1^, in effort to further increase line width. This change was marginally successful and increased the printed line width to 37 μm. Albeit, the increased dwell time at a given position resulted in a slight increase in overspray. The effect of print speed was more thoroughly analyzed with regards to printed line width, thickness, and profile in a subsequent study, Figure S1, where the resultant lines were characterized using a PIAS-II system integrated with ASTM ISO 13660 standards^45–48^.

Analysis from the 6-h drift study showed that the line quality had improved over the course of the production run. This result is in slight disagreement with the existing literature on instrumental drift^44^, which shows a decrease in print quality after a certain duration of printing. We speculate that initial ink volume influences the drift behavior of the system, and increasing this value may effectively extend the ideal processing window at the cost of an increased upstart time to reach system equilibrium. This helps to explain why a time interval showing decreased print quality was not observed during the 6-h print session. While the reason for this drift is not totally clear, the results from the drift profile can be used to inform future prints and help to improve consistency in fabrication quality and predictability of outcome.

The residual ink was collected after printing for rheological characterization. DLS was performed on the residual ink and the effective diameter of the silver nanoparticles had decreased from the initial measurement of 90 nm to 45 nm, measured immediately after printing. This preliminary result indicates that the ink had improved in quality over the course of the print session. We originally hypothesized that the inks prolonged exposure to ultrasonic energy from the atomizer served to break apart soft agglomerations suspended in the ink that accumulate during storage. However, a study was performed in which the print system was disassembled at selected time intervals to retrieve aliquots for particle size analysis, Figure S2. The results from this experiment showed little to no change over the course of a 6-h print session and only a minor decrease in average hydrodynamic particle size between hour 0 and hour 1 with regards to hours of atomizer exposure. Based on these results, we have revised our hypothesis and plan to perform studies examining particle loading of the ink over time, investigating ink stability and particle sedimentation as a factor in instrumental drift.

## Results and discussion

### Electrical devices

#### Design and sintering

During the initial print session, silver devices were fabricated on glass for structural and electrical characterization. The devices were designed to have a 40 μm printed line width and a serpentine fill pattern using a line overlap of 50–60%. 4-point devices were designed to have an effective line dimension of 3 × 0.5 mm, connected to four 1 mm^2^ contact pads. A 1 cm square was printed, using similar fill parameters, for grain size analysis and to determine the degree of grain growth that occurs using the material supplier’s recommended sintering conditions.

X-ray diffraction (XRD) was first performed on the unsintered sample using a Rigaku miniflex X-ray diffractometer. In this experiment, a copper source was used to generate the X-rays (1.541 Å), and the sample was measured in the scan range of 2*θ* = 10^∘^–70^∘^. The primary peak position of the printed silver thin film was observed at 2*θ* = 39. 1^∘^ and is related to the crystal plane (111). Secondary diffraction peaks are observed at 2*θ* = 44. 3^∘^ and 64. 5^∘^, which correspond to the (200) and (220) crystal planes in silver. A broad peak can be observed at 2*θ* ≈ 25^∘^, which is indicative of glass stretching and was dismissed from the analysis. The full width half maximum of the primary peak in the unsintered silver sample was determined to be *β* = 0.447^∘^, relating to a grain size of *τ* = 20.6 nm, calculated using the Scherrer equation, shown as Equation (4).

4 $$\tau =\frac{k\lambda }{\beta \cos (\theta )}$$

**Fig. 4:**
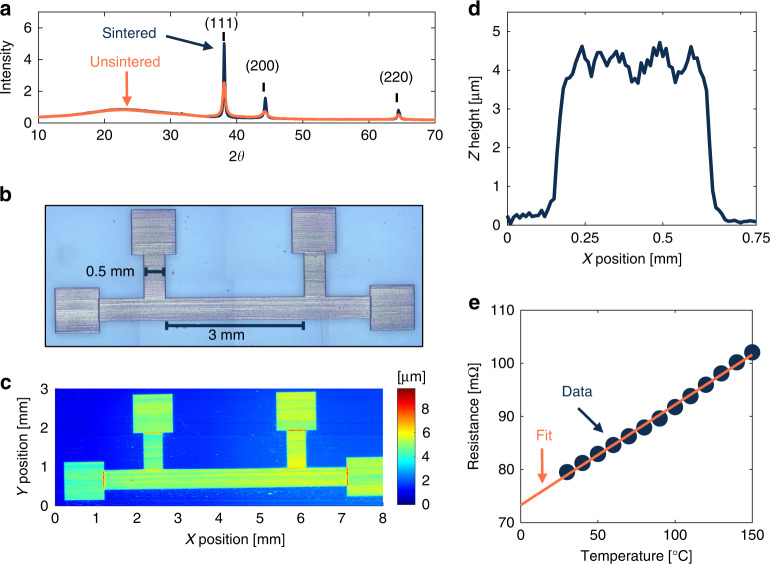
Structural and electrical properties of printed silver devices. **a** X-ray diffraction spectra of Clariant silver nanoparticle ink deposited on a glass slide, before and after sintering for 1 h at 200 ^∘^C. Crystal planes of the silver film are labeled above the respective peaks. **b** Dimensionally annotated stitched microscope image of the printed 4-point device for electrical characterization. **c** Stylus profilometry map of the printed 4-point structure. **d** Mean line profile of the effective line in the 4-point device. **e** Resistance vs. temperature plot of the printed silver 4-point structure

Here, *k* is a dimensionless shape factor dictated by particle morphology, *λ* is the wavelength of X-rays emitted from the source, *β* represents the full width at half maximum of the primary peak, and *θ* corresponds to the angle between the reflecting plane of the crystal and the incident beam. Instrumentation broadening was considered and subtracted off prior to performing the grain size calculations. The printed sample was sintered in the oven at 200 ^∘^C for 1 h. Collection of the XRD data for the sintered sample revealed that the grain size of the silver nanoparticles increased to *τ* = 60.7 nm, resulting in a Δ*τ* = 40.1 nm due to the sintering process, Fig. 4a.

Cross-sectional analysis was performed on both unsintered and sintered silver lines, printed with the settings described in this paper, Figure S3. The total projected area of the unsintered lines was determined determined to be 154.8 μm^2^ and the sintered lines was 120.4 μm^2^. Therefore, the printed lines were densified by 22.2% upon sintering. Cross-sectional porosity was determined using a 35% threshold low mask and showed a relative cross-sectional porosity of 9.5%.

#### Profilometry and electrical characterization

Figure 4b is a stitched microscope image of a printed 4-point structure, dimensionally annotated with the design parameters. 3D scanning was performed on the printed samples using a Bruker Dektak stylus profilometer to generate a map of the surface, Fig. 4c. Maps of the stylus profilometry data were generated using Vision 64 software, and analysis of the data was performed using MATLAB. The 4-point line profile, Fig. 4d, was averaged over the full length of the effective line to maximize the accuracy of the cross-sectional area calculation. Using the printing parameters in Table 1 and the fill parameters described in this section resulted in a line profile z-height ≈ 4.2 μm. Integration of the average line profile revealed a cross-sectional area, *A* = 2095 μm^2^.

A Cascade microprobe station was used to initiate contact with the sample, and the temperature was controlled using a Temptronic heating chuck. Resistance measurements were performed on the printed devices using a Keithley 6221/2182A current source and nanovoltmeter combo. The dependence of the sensor resistance as a function of temperature was monitored using a 4-point Delta Mode technique^49^. Resistance of the 4-point structure was measured in 10 ^∘^C steps from 30 to 150 ^∘^C, Fig. 4e. From these measurements, the temperature coefficient of resistance *α* was calculated using Equation (5) and the resistivity of the printed thin film (*ρ*) was calculated using Equation (6).

5 $$\alpha =\frac{R-{R}_{0}}{{R}_{0}(T-{T}_{0})}$$

6 $$\rho =\frac{RA}{L}$$

*L* represents the length of the effective line, *R* is the resistance measured at temperature *T*, and *R*_0_ is the resistance measured at *T*_0_. The temperature coefficient of resistance was calculated to be ${{{\rm{\alpha }}}}=2.36\times 1{0{}^{-3}}^{\circ }$C^−1^ and the resistivity was calculated to be *ρ* = 5.38 × 10^−8^Ωm. Comparing the resistivity of the printed thin film to the value of bulk silver yields *ρ*_0_/*ρ* ≈ 0.3, therefore the printed thin films exhibited a conductivity of 3.4 × less than bulk. This metric is used in the following section for quality factor evaluation of the printed lines in the IDT structures.

### Surface acoustic wave device

#### Design and profilometry

**Fig. 5:**
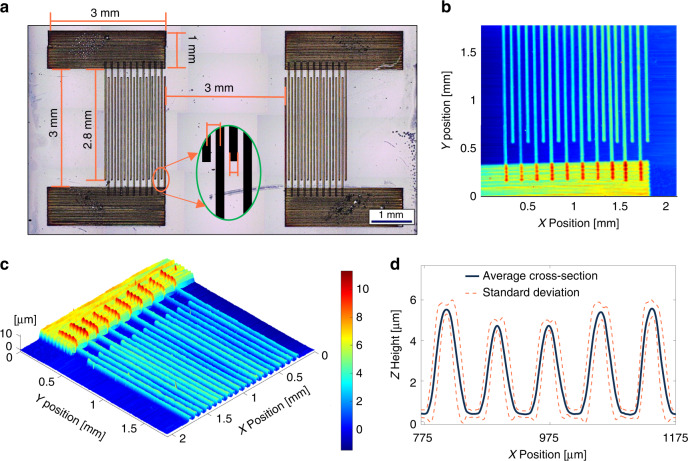
Design and profile of the printed SAW device. **a** Stitched microscope images of a printed SAW device annotated with the dimensions. Inset image defines effective width and pitch of electrodes. **b**, **c** Stylus profilometry map of printed SAW device. **d** Average height and width of printed electrode fingers from 3D scans

The process parameters presented in Table 1 were used to fabricate a SAW thermometer consisting of two IDTs printed on a 10 × 10 × 0.5 mm SAW-grade single crystal LiNbO_3_ substrate, Fig. 5a. For each set of comb electrodes a 3 × 1 mm^2^ electrode pad was fabricated to facilitate SAW thermometer integration. Driven by a pulsed voltage, one IDT (transmitter) generates an impulsive SAW propagating towards the other IDT (receiver), where the SAW is converted back to an electrical signal. As temperature increases, the LiNbO_3_ softens, leading to a reduced speed of sound and an increased travel distance due to thermal expansion. Therefore, LiNbO_3_-based SAW devices can detect ambient temperature by tracking time-of-flight between IDTs^50^. However, time-of-flight measurement is not only sensitive to surrounding noise, but also requires an excessively high sampling rate or long distance between IDTs to achieve small measurement resolution.

A more convenient and accurate method to extract temperature information from a LiNbO_3_-based SAW device is to measure the thermal drift in its resonant frequencies. The fundamental frequency of a SAW device is described by Equation (2), and the thermal drift in fundamental frequency follows

7 $$\frac{{{\Delta }}f}{{{\Delta }}T}=\frac{f(T)-{f}_{r}}{T-{T}_{r}}=\frac{1}{{l}_{w}}\frac{\partial {V}_{{\mathrm{SAW}}}}{\partial T}-{\alpha }_{p}\frac{{V}_{{\mathrm{SAW}}}}{{l}_{w}^{2}}\approx \frac{1}{{l}_{w}}\frac{\partial {V}_{{\mathrm{SAW}}}}{\partial T},$$

where Δ*T* is the temperature increment relative to room temperature *T*_*r*_, *f*(*T*) is the fundamental frequency of the printed SAW thermometer at temperature *T*, *f*_*r*_ = *f*(*T*_*r*_), and *α*_*p*_ is the coefficient of thermal expansion of LiNbO_3_. The impact of thermal expansion is about two orders of magnitude lower than that of the temperature-induced speed of sound reduction, and therefore, can be neglected^51,52^. The SAW thermometer sensitivity is defined as

8 $${S}_{{\mathrm{SAW}}}=\frac{f(T)-{f}_{r}}{{f}_{r}}\cdot \frac{1}{{{\Delta }}T}.$$

According to Equation (1), (7), and (8), *S*_SAW_ depends on IDT geometries, including the total number of finger pairs *n*_*f*_ and the metallization ratio *M* = *w*_*f*_/*p*_*w*_, where *w*_*f*_ is the electrode width and *p*_*w*_ is the pitch of IDTs or the distance between two adjacent electrodes, Fig. 5a inset. As *n*_*f*_ increases, the resonant peak narrows and measurement accuracy improves. Limited by the substrate size, *n*_*f*_ = 10 was selected for this study. A higher *M* will increase the mass loading effect, reduce SAW resonance, and decrease resonant peak amplitude. On the other hand, adjustment of *M* is an effective approach to obtain 50-Ohm impedance match for sensor integration^53^. To simplify data analysis, a *M* = 0.5 or *l*_*w*_ = 4*w*_*f*_ was targeted for this study.

A quick processing of the microscopic images, Fig. 5a, shows that *w*_*f*_ ≈ 37 μm and *M* ≈ 0.465 for the printed silver IDTs. 3D scanning was performed on the sample to collect a more accurate 3D surface topography of the printed IDTs, Fig. 5b. Using the fast Fourier transform to process the topography data at three separate cross-sections of the device, Fig. 5d, revealed the average *l*_*w*_ = 159.2 μm, which corresponded to a *f*_*r*_ of 21.9 MHz. The surface topography results established that the average electrode height generated from these settings is 5.29 ± 0.54 μm, and the full width half maximum of the electrode fingers was measured at 46 individual cross-sections of the device, Fig. S4. The average FWHM was calculated to be *β* = 32.13 ± 2.24 μm. The quality factor of the printed electrodes was calculated, as described by Smith et al., to benchmark the print quality of these process parameters against existing literature.

9 $${\mathrm{QF}}=\frac{\beta }{{w}_{f}}\times \frac{{\rho }_{0}}{\rho }$$

This metric considers both the printed line quality and the electrical performance of the printed device, represented in Equation (9) as the width ratio (*β*/*w*_*f*_) and relative conductivity (*ρ*_0_/*ρ*), respectively^40^. The quality factor of the printed SAW electrodes was calculated to be QF = 0.261 ± 0.018. This result is on par with the existing literature concerning QF of AJ-printed lines. It was concluded that the printer settings outlined in this paper produced features with an improved width ratio, however, the relative conductivity of the printed SAW device is lower than reported. By improving the sintering conditions in future studies, the feature quality produced by these process parameters can be even further improved^54^.

#### SAW characterization

The printed SAW thermometers were eventually characterized on a Cascade probe station equipped with a heating chuck capable of reaching 200 ^∘^C. Although LiNbO_3_ has a relatively high Curie point (1140 ^∘^C), it has been shown to undergo thermal degradation beginning at temperatures greater than 300 ^∘^C^55^. The fundamental frequency *f*(*T*) was extracted from scattering parameter measurements provided by a vector network analyzer, including the reflection coefficient *S*_11_ across the transmitter (port 1) and the forward voltage gain *S*_21_ between the transmitter and receiver (port 2). Values of *S*_11_ and *S*_21_ describe the SAW thermometer working in the pulse-echo mode and the pulse-receiver mode, respectively. At resonances, *S*_21_ will maximize and *S*_11_ will minimize, which indicates that the amount of energy transferring from the transmitter to the receiver is maximized.

**Fig. 6:**
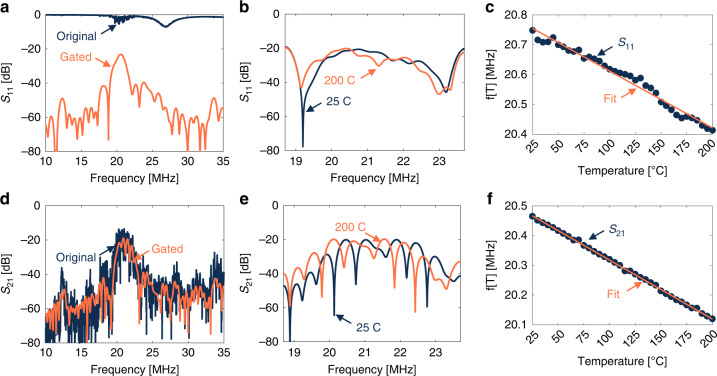
Characterization and temperature response of AJ-printed surface acoustic wave device. **a** *S*_11_ and (**d**) *S*_21_ measurements at room temperature before and after time gating. Time-gated (**b**) *S*_11_ and (**e**) *S*_21_ measurements at room temperature and 200 ^∘^C. Thermal drift of fundamental frequency *f*(*T*) obtained from gated (**c**) *S*_11_ and (**f**) *S*_21_ as ambient temperature increases from 25 to 200 ^∘^C in 5 ^∘^C steps

For characterization of the SAW devices, one set of the comb electrodes was grounded through a high-performance DC paramatric probe that is rated for > 100 MHz measurements. The other set was wired to a vector network analyzer (Copper Mountain TR 1300/1) using the same type of probe. A coarse search was performed over a range of *f*_*r*_ between 10 and 35 MHz, Fig. 6(a, d). Scattering parameters are extremely noisy due to the excessive acoustic reflections from the edges of LiNbO_3_ substrate and the electromagnetic coupling between IDTs. In addition, an unexpected valley in *S*_11_ was observed at around 27 MHz, which interfered with the resonant response at *f*_*r*_. We suspect that this valley originates from the silver pads dynamics. This unexpected valley consistently appeared in all four SAW thermometers that were printed. Time domain gating was therefore used to correct the scattering parameter measurements. The time window for *S*_11_ correction is from 5 to 6.5 μs. In this case, the receiver works as a mechanical filter that only reflects the fundamental component of the SAW. Once time-gated, the unexpected valley at 27 MHz was removed, and no longer influenced the measurement of the *S*_11_ peaks at *f*_*r*_. The time window for *S*_21_ correction is from 1 to 3.5 μs, covering both the SAW arrival and departure at the receiver. The time windows used in this study were estimated using a COMSOL Multiphysics model. Figure 6(a, d) confirmed the effectiveness of time gating.

Prior to thermometer characterization, two Type-K thermocouples were mounted on the heating chuck and a similar LiNbO_3_ substrate, in order to quantify temperature stability and distribution. This preliminary experiment confirmed that both the heating chuck and the substrate temperatures stabilized within 2 min, and therefore, data acquisition was not started until at least 3 min after the temperature controller had reached the temperature set-point. Probe tips were lifted before increasing the temperature of the device to avoid damage to the contact pads by thermal expansion. Scattering parameters were collected and post-processed using the TRVNA software from Copper Mountain.

Owing to constraints by the buffer size of the vector network analyzer (16,001 data points), the frequency sweep range was narrowed for subsequent measurements to 18.7–23.7 MHz, Fig. 6(b, e). The frequency resolution is *f*_step_ = 312.5 Hz, resulting in a temperature measurement resolution of *T*_step_, where

10 $${T}_{{\mathrm{step}}}=\frac{{f}_{{\mathrm{step}}}}{{f}_{r}\parallel {S}_{{\mathrm{SAW}}}\parallel }.$$

Five measurements were taken at each temperature to quantify uncertainties in frequency measurement *δf*. The corresponding measurement uncertainty in temperature is,

11 $$\delta T=\frac{\delta f}{{f}_{r}\parallel {S}_{{\mathrm{SAW}}}\parallel }.$$

We used the linear regression method to fit the temperature varying *S*_11_ and *S*_21_ (Fig. 6(c, f). Sensor linearity is described by the coefficient of determination.

As shown in Table 2, the AJP-printed SAW thermometer exhibited superior linearity and repeatability in both the pulse-echo mode and the pulse-receiver mode. The *S*_SAW_ derived from *S*_11_ and *S*_21_ are 2.0% and 3.2% away from the nominal value (95 × 10^−6^/^∘^C) reported in literature^37^. Overall, measurement repeatability is slightly better in the pulse-echo mode, because the acoustic signal is filtered by the receiver and the resonant peak identification is more accurate. However, the pulse-receiver mode delivers a better linearity because *S*_21_ data exhibits a greater signal-to-noise ratio.

**Table 2:** Performance of AJ-printed SAW thermometer

Measurement	*S*_11_	*S*_21_
Sensor mode	Pulse-echo	Pulse-receiver
Sensitivity ($\times 1{0{}^{-6}}^{\circ }$C^−1^)	-96.9	-92.0
Average uncertainty ($\times 1{0{}^{-10}}^{\circ }$C)	6.73	31.00
Coefficient of determination (-)	0.978	0.999
Resolution ($\times 1{0{}^{-7}}^{\circ }$C)	1.55	1.66
*f*_*r*_ (MHz)	20.75	20.48

## Conclusion

We have successfully devised a 20 MHz SAW thermometer using AJP to deposit silver IDTs onto Y-cut SAW-grade lithium niobate substrates. Rheological properties of the ink were measured to ensure compatibility with the AJP system. Printer settings were chosen experimentally, but informed by existing literature. Targeting a line width of *w*_*f*_ = 40 μm, the gas flow settings were determined by adjusting the focusing ratio and print speed from established printer settings^39^. The working distance was determined from the calculated Reynold’s number of the system^43^. Coating thickness for the printing parameters was measured at different speeds to increase the utility of these settings. The print quality of the lines was observed at different time intervals to investigate the degree of instrumental drift, showing marketable improvements as time passed in the study. Establishing a process window of high-quality fabrication with respect to time has served to improve both the consistency and predictability of outcome in our fabrication process. It was also our observation that some rheological properties of the ink had changed over time throughout the 6-h print. Our current hypothesis is that the particle loading in the nanoparticle silver ink changes over time and eventually affects the printing quality. We speculate that increasing the initial ink volume for AJP prolongs the ideal processing window, but may also be related to a longer upstart time to reach equilibrium status for the system. Printed samples in this study were optically and mechanically profiled to determine structural aspects of the devices and to provide data for the resistivity and quality factor calculations. Measurement of the printed structures using the AJP settings presented in this paper resulted in a z-height of 5.3 μm for printed lines, and 4.2 μm for pad filling operations. Cross-sectional SEM was performed on the printed lines before and after sintering, showing a densification of 22.2% and a cross-sectional area of 120.4 μm^2^. Printed devices were tested structurally and electrically, revealing a Δ*τ* = 40.1 nm due to sintering, and a *ρ* of 3.4 × greater than bulk silver. The quality factor of the printed lines was determined to be QF = 0.261 ± 0.018. We experimentally validated the SAW thermometer by measuring *S*_11_ and *S*_21_ from 25 to 200 ^∘^C. Sensitivities of the printed SAW thermometer are $-96.9\times 1{0{}^{-6}}^{\circ }$C^−1^ and $-92.0\times 1{0{}^{-6}}^{\circ }$C^−1^ when operating in the pulse-echo mode and the pulse-receiver mode, respectively. Time gating was performed on the printed sensors to improve signal reliability and to reduce noise.

## Supplementary information

Supplementary Material
