# Supplementary material for: Aerosol jet printing of piezoelectric surface acoustic wave thermometer
Source: Microsyst Nanoeng. 2023 May 4;9:51. doi: 10.1038/s41378-023-00492-5 (PMC10159840; doi:10.1038/s41378-023-00492-5)
Supplement: Supplementary file 1 — Supplementary Material [file 41378_2023_492_MOESM1_ESM.pdf]

6 Supplementary Material

6.1 Print Quality Analysis

Subsequent studies were performed using the printer settings outlined in the main-text to further elucidate the effect of variation in process parameters. In this study, repeating serpentine lines were printed targeting 40 μm width with 40 μm spacing. This 80 μm pitch design was utilized to emulate the reported design of the IDTs. The square serpentine line pattern was 8x8 mm^2, therefore 100 printed lines with 100 spaces were printed per run, with print time being determined by printer speed. Silver lines were deposited at speeds in the range of 0.25 - 4.0 $mm \cdot s^{-1}$. The printed lines were characterized using stylus profilometry which provided the line profile data, **Figure** S1a and the coating thickness data for the printed lines, Figure S1c.

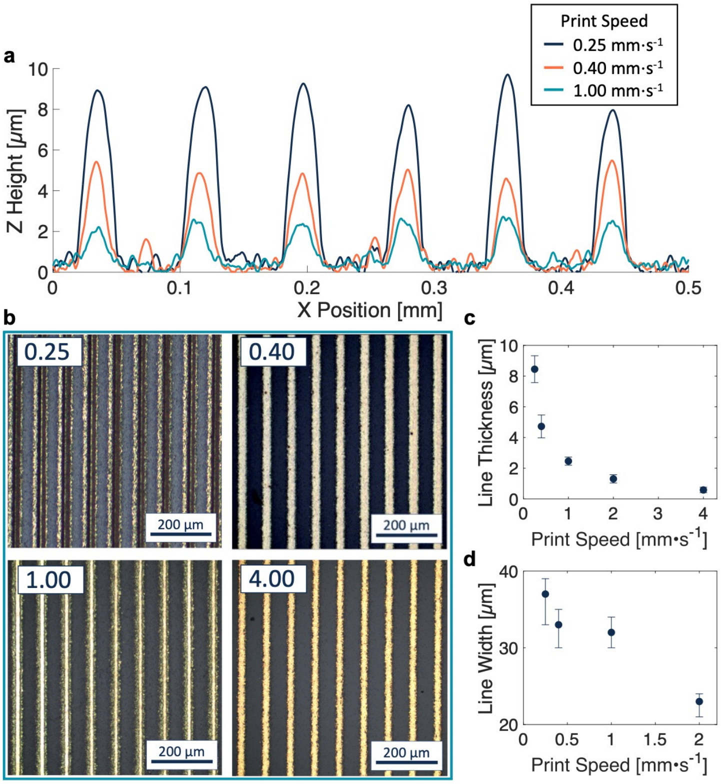

Fig. S1 (a) Profile measurements of printed silver lines deposited at varying print speeds and overlaid for viewability. (b) Microscope images show lines with different print speeds. (c) Average coating thickness measurements generated from 2 seperate cross-sections of the serpentine patterns. (d) Mean width measurements from PIAS-II system, automatically generated from data sets of 20+ printed lines.

Image analysis, both manual or automated, is predominant in print quality characterization of printed devices [1,2]. In this study we utilized the PIAS-II (QEA Inc) print quality analysis tool to measure the printed line width. This instrument measures line edge as a percent reflectance,[3] in accordance with ISO-13660 standards [4]. The same standards can be applied to AJP printed lines since they are device independent. Measurement of the printed lines from this study showed that print speed is a potentially effective way to tune the printed line width, Figure S1d.

6.2 Rheological Drift

We performed a hydrodynamic particle size vs. atomization time study on the silver nanoparticle ink to further investigate an observed decrease in particle size over print time.

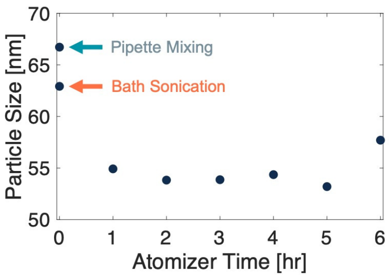

Fig. S2 Hydrodynamic particle size vs. atomizer exposure time.

In this experiment, the print system was disassembled at selected time intervals and a 20 µl aliquot of ink was collected and diluted in 10 ml of water for characterization. The results from this experiment showed little to no change over the course of a 6- hour print session and only a minor decrease was observed in average hydrodynamic particle size between t_0 and t_1. It should be noted that the time between experimental sets was 1 year, and the Clariant suspension had reached its expiration date. While we found it to be still usable for manufacturing, some of the ink properties had changed, which served to convolute the study.

6.3 Cross-sectional Analysis

Scanning electron microscopy was performed on the printed serpentine lines to determine printed line width, cross-sectional area, degree of consolidation, porosity, etc. Cross-sectional imaging of the deposited silver lines was performed using an FEI Teneo Field Emission Scanning Electron Microscope FESEM. SEM Samples were prepared by cleaving the glass substrates and were carbon coated before sample evacuation. High vacuum was used for imaging and EDS elemental analysis. The ETD and T1 detectors were used to obtain varying resolutions in imaging the deposited material.

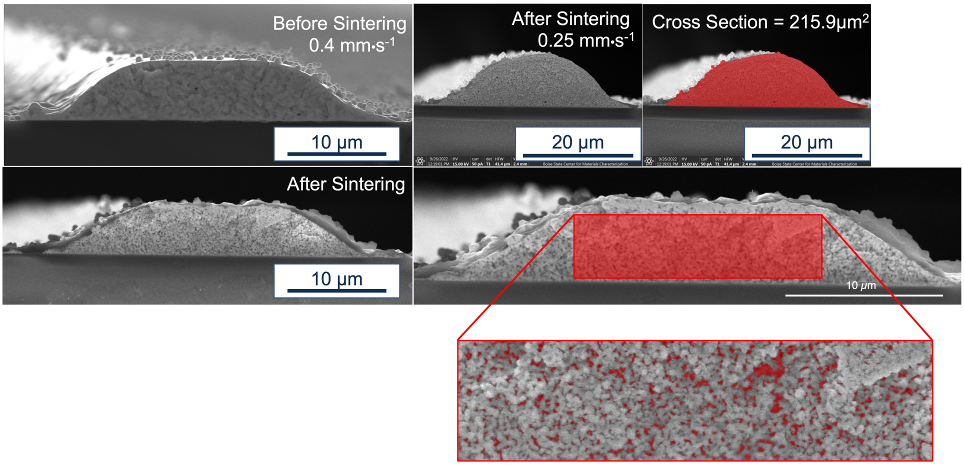

Fig. S3 Cross-sectional SEM images of printed silver lines. For a print speed of 0.4 mm*s$^-$1, the cross-sectional of the printed line was determined to be 120.4 µm^2 after sintering, showing a densification of 22.2% and a cross-sectional porosity of 9.5%.

The sintering process of the deposited silver has a significant effect on the morphology of the lines, reducing the line height from about 6.0µm to 5.0µm, **Figure** S3. Samples printed at the speed of 0.4 $\mathrm{mm} \cdot \mathrm{s}^{-1}$ showed a densification of 22.2% due to the thermal processing conditions described in the primary article. This densification of the film comes from the evaporation of solvents left over after deposition and the sintering of the nanoparticles into a conductive film. The porosity of the film was analyzed using Gwyddion software to threshold map the SEM images of the film cross-section. The mask was set to 35% low threshold, and the relative area function was used to find percentage porosity. Red masks are displayed over the pores in the film after sintering, and the porosity was calculated to be 9.5%.

Non-destructive cross-sectional analysis was performed on the printed SAW device to determine the consistency of the printed electrodes. 46 different cross-sections were analyzed from the profile map (every 25 μm, totaling 920 data points). The average cross-sectional area was measured to be Aσ = 204.42 $\pm$ 8.93 μm^2 from stylus profilometry.

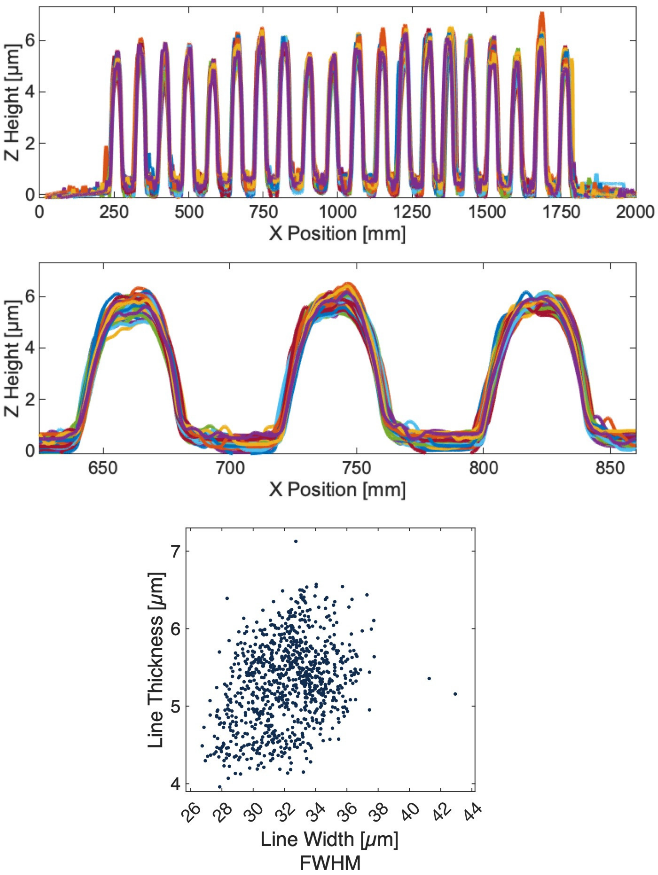

Fig. S4 Cross-sectional analysis of the printed SAW device from stylus profilometry, and scatterplot showing clustered region of the printed line widths.

6.4 Supplementary References

(1) Zhang, H., Moon, S. K. & Ngo, T. H. Hybrid Machine Learning Method to Determine the Optimal Operating Process Window in Aerosol Jet 3D Printing. ACS Applied Materials and Interfaces 11, 17994-18003 (2019). https://doi.org:10.1021/acsami.9b02898

(2) Zhang, H., Choi, J. P., Moon, S. K. & Ngo, T. H. A multi-objective optimization framework for aerosol jet customized line width printing via small data set and prediction uncertainty. Journal of Materials Processing Technology 285, 116779-116779 (2020). https://doi.org:10.1016/j.jmatprotec.2020.116779

(3) Briggs, J. C., Klein, A. H. & Tse, M.-K. Applications of ISO-13660, a new international standard for objective print quality evaluation. Japan Hardcopy 99, 21-23 (1999).

(4) Barney Smith, E. Relating Electrophotographic Printing Model and ISO13660 Standard Attributes. Elisa H. Barney Smith 7529 (2010). https://doi.org:10.1117/12.841770
